# Alcohol Ablation for Cardiac Arrhythmias: Is it Time to Drown the Arrhythmias?

**DOI:** 10.1016/s0972-6292(16)30520-4

**Published:** 2012-07-28

**Authors:** Anandaraja Subramanian

**Affiliations:** Consultant Cardiology and Electrophysiologist, IGGGH & PGI, Pondicherry, India

**Keywords:** Catheter ablation, alcohol ablation, ventricular arrhythmias

Radiofrequency catheter ablation (RFCA) is the treatment of choice for cardiac arrhythmias. However, sometimes, RFCA may be insufficient to eliminate the arrhythmias. Arrhythmic foci that are deep seated in the myocardium may not be amenable to catheter ablation from either the endocardium or the epicardium. Catheter ablation may also fail to produce a complete lesion and there can be surviving myocardial cells that sustain the arrhythmia. Alcohol ablation (AA) of coronary artery offers an alternative option in such cases. Alcohol ablation produces homogenous tissue lesion when applied to the target sites.

Alcohol ablation for arrhythmias was reported first in 1987 by Inoue et al in dogs [1]. The procedure has been reported in the literature sporadically since then with first clinical report in 1988 by Brugada et al [2]. The largest experience has been reported recently by Tokuda et al [3] for the treatment of ventricular arrhythmias. Though the procedure had relatively early beginning, it failed to take off like RF ablation because of safety concerns and steep learning curve. Alcohol ablation of coronary artery has been extensively used in the treatment of hypertrophic cardiomyopathy [4].

Currently alcohol ablation is advocated as an alternative option in the treatment of cardiac arrhythmias. Failure to ablate the arrhythmias either by endocardial or epicardial route is an indication for alcohol ablation. Alcohol ablation has been successfully used in the ablation of ventricular tachycardia [2,5,6], ventricular fibrillation [7] and atrial fibrillation [8]. In this issue of the Journal, Roten L et al [9^9^] have reported the use of alcohol ablation for successfully terminating an upper septal ventricular tachycardia. Success could not be achieved with endocardial and epicardial routes. Injection of absolute alcohol in the first septal branch created an area of necrosis in the septum that abolished the arrhythmic foci in the septum. Though the authors had successful ablation without any procedural complication, about one third of the patients can develop AV block requiring permanent pacemaker implantation when septal artery was the target vessel [3]. However, alcohol ablation can be safely performed when the target area is away from the septum. Overall, AA was effective in controlling and preventing recurrences of arrhythmias in about two third of the cases.

With the exciting and promising results, can AA be used as a first line option in the treatment of septal tachycardias or scar related tachycardias away from the upper septum? Alcohol ablation is not recommended in patients with normal heart and idiopathic arrhythmias for fear of creating an arrhythmic substrate. Currently all reported cases and series have only included patients with myocardial scar. In some cases, in spite of good candidates, alcohol ablation may not be feasible because of absent target vessel to the arrhythmic site or the vessel having collateral supply to adjacent myocardium. So a good strategy will be to perform initial endocardial ablation and epicardial ablation before adopting AA. In case of postoperative patients, pericardial space can be difficult to access and AA can be attempted after failed endocardial ablation. Though it is tempting to attempt an initial AA for septal tachycardia, because of the risk of heart block, it is prudent to start with endocardial ablation. Incidence of atrioventricular block is reported to be as high as 66% and roughly half of them will require permanent pacemaker implantation [3].

To conclude AA is an excellent alternative option in selected cases. Arrhythmias originating from deep myocardial location, arrhythmias refractory to endocardial and epicardial ablation can be offered alcohol ablation. The procedure has good success rate with definite complications. Till the techniques are perfected and we gain sufficient experience, the procedure has to be used with caution.
